# Oligo-carrageenan kappa increases NADPH, ascorbate and glutathione syntheses and TRR/TRX activities enhancing photosynthesis, basal metabolism, and growth in *Eucalyptus* trees

**DOI:** 10.3389/fpls.2014.00512

**Published:** 2014-10-13

**Authors:** Alberto González, Fabiola Moenne, Melissa Gómez, Claudio A. Sáez, Rodrigo A. Contreras, Alejandra Moenne

**Affiliations:** Marine Biotechnology Laboratory, Department of Biology, Faculty of Chemistry and Biology, University of Santiago of ChileSantiago, Chile

**Keywords:** ascorbate, basal metabolism, *Eucalyptus globulus*, glutathione, NADPH, redox status, thioredoxin reductase, thioredoxin

## Abstract

In order to analyze the effect of OC kappa in redox status, photosynthesis, basal metabolism and growth in *Eucalyptus globulus*, trees were treated with water (control), with OC kappa at 1 mg mL^−1^, or treated with inhibitors of NAD(P)H, ascorbate (ASC), and glutathione (GSH) syntheses and thioredoxin reductase (TRR) activity, CHS-828, lycorine, buthionine sulfoximine (BSO), and auranofin, respectively, and with OC kappa, and cultivated for 4 months. Treatment with OC kappa induced an increase in NADPH, ASC, and GSH syntheses, TRR and thioredoxin (TRX) activities, photosynthesis, growth and activities of basal metabolism enzymes such as rubisco, glutamine synthetase (GlnS), adenosine 5′-phosphosulfate reductase (APR), involved in C, N, and S assimilation, respectively, Krebs cycle and purine/pyrimidine synthesis enzymes. Treatment with inhibitors and OC kappa showed that increases in ASC, GSH, and TRR/TRX enhanced NADPH synthesis, increases in NADPH and TRR/TRX enhanced ASC and GSH syntheses, and only the increase in NADPH enhanced TRR/TRX activities. In addition, the increase in NADPH, ASC, GSH, and TRR/TRX enhanced photosynthesis and growth. Moreover, the increase in NADPH, ASC and TRR/TRX enhanced activities of rubisco, Krebs cycle, and purine/pyrimidine synthesis enzymes, the increase in GSH, NADPH, and TRR/TRX enhanced APR activity, and the increase in NADPH and TRR/TRX enhanced GlnS activity. Thus, OC kappa increases NADPH, ASC, and GSH syntheses leading to a more reducing redox status, the increase in NADPH, ASC, GSH syntheses, and TRR/TRX activities are cross-talking events leading to activation of photosynthesis, basal metabolism, and growth in *Eucalyptus* trees.

## Introduction

The redox status in plants is mainly determined by the levels of NADPH, NADH, ascorbate (ASC), and reduced glutathione (GSH) as well as by the activities of antioxidant enzymes using NADPH, NADH, ASC, and GSH as substrates (Foyer and Noctor, 2011). NADH/NADPH are essential reducing compounds for plant viability since the disruption of the unique gene encoding nicotinamide mononucleotide adenylyl transferase (NMNAT), a key regulatory enzyme of NAD synthesis, was lethal in *Arabidopsis thaliana* (Hashida et al., 2009). In addition, the heterozygous mutant of *nmta* gene showed shorter siliques with lower seed set indicating that NAD(P)H content may be involved in regulating growth and development (Hashida et al., 2007). ASC is also an essential molecule for plant viability since *Arabidopsis* mutants completely deficient in ASC were observed to be seedling lethal (Dowdle et al., 2007). In addition, it has been shown that ASC is essential for transition from G1 to S phase as recorded in tobacco cells cultured *in vitro* as well as in cells of the quiescent center in onion roots (Liso et al., 1988; De Pinto et al., 1999). Furthermore, ASC may regulate growth since tomato mutants having a lower activity of L-galactonolactone dehydrogenase (GLDH), the last and regulatory enzyme for ASC synthesis, showed a decrease in growth rates, and in leaves and fruit sizes (Alhagdow et al., 2007). In addition, GSH is essential for plant viability since mutants deficient in GSH synthesis were observed to be embryo lethal (Cairns et al., 2006). Moreover, GSH was observed to be necessary for transition from G1 to S phase in tobacco cells cultured *in vitro*, and for cell division in the apical meristem of *A. thaliana* roots (Vernoux et al., 2000). Furthermore, GSH may also regulate photosynthesis and growth since cucumber plant treated with a brassinosteroid showed an increased in GSH/GSSG ratios followed by an increased photosynthesis and C assimilation (Jiang et al., 2012). In addition, *A. thaliana* individuals having a mutation in the gene encoding for γ-glutamylcysteine synthase (γ-GCS), the first and regulatory enzyme involved in GSH synthesis, and in NADPH-dependent thioredoxin reductases (TRRs) showed smaller size shoots and roots and a decrease in auxin levels, and in the expression of genes encoding auxin transporters (Bashandi et al., 2010).

In addition, plant growth is also regulated by the coordinated assimilation of carbon (C), nitrogen (N), and sulfur (S), which are reductive light-dependent processes (Kopriva et al., 1999, 2002; Lillo, 2008; Kraiser et al., 2011; Takahashi et al., 2011). Carbon assimilation is partly subject to photosynthesis and activities of Calvin-Benson cycle enzymes, mainly by ribulose 1,5-biphosphate carboxylase/oxygenase (rubisco) that catalyzes the limiting reaction responsible of reducing atmospheric CO_2_ into a three-carbon sugar (Kopriva et al., 2002). Nitrogen assimilation requires the uptake of nitrate from the soil, which is reduced to nitrite and then to ammonium in the shoots where, in turn, takes part of the aminoacids glutamine and glutamate (Kraiser et al., 2011). The latter aminoacids are synthesized by the enzymes glutamine synthetase (GlnS) and glutamate dehydrogenase (GDH), respectively, and GlnS activity is the limiting reaction. On the other hand, sulfur assimilation involves sulfate uptake from the soil which is reduced to sulfite and then to sulfide in the shoots where, in turn, takes part of the aminoacid cysteine after an O-acetylserine thiol-lyase catalyzed reaction (O-ASTL) (Takahashi et al., 2011). Moreover, the reduction of adenosine 5′-phosphosulfate (APS) to sulfite is mediated by the enzyme adenosine 5′-phosphosulfate reductase (APR), which is the limiting reaction in sulfur assimilation (Vauclaire et al., 2002).

In addition, it is well known that NADPH levels are involved in regulating basal metabolism through TRRs/thioredoxins (TRXs) system, since TRR uses NADPH as substrate, TRRs reduce TRXs, and TRXs reduce disulfide residues of many key metabolic enzymes inducing their activation and, only in few cases, their inactivation. Moreover, TRR/TRX activities have been shown to influence photosynthesis, C, N and S assimilation and growth (Gelhaye et al., 2005; Montrichard et al., 2009). In particular, TRXs interact with the small and the large subunits of rubisco and rubisco activase (RAC), an ATP-dependent chaperone that removes sugar-phosphates from rubisco's active site (Motohashi et al., 2001; Gelhaye et al., 2005). In addition, functional assays have demonstrated that TRXs activate RAC which, in turn, increase rubisco activity in *A. thaliana* (Zhang and Portis, 1999). Furthermore, TRXs bind to GlnS, the regulatory enzyme of N assimilation (Yamazaki et al., 2004), and mediate the increase in GlnS and glutamate synthase (GluS) activities in the green microalga *Chlorella sorokiniana* (Tischner and Schmidt, 1982). Until now, there is no evidence indicating that TRXs are directly involved in the activation of APR and O-ASTL, but it has been recently shown that TRXs activate a cyclophilin, which is a molecular chaperone with peptidyl prolyl isomerase activity, that activates O-ASTL in *A. thaliana* (Dominguez-Solís et al., 2008). Until now, there is no evidence demonstrating that TRR/TRX system is involved in the regulation of NAD(P)H, ASC and/or GSH syntheses.

In previous work, we determined that oligo-carrageenans (OCs), obtained by acid depolymerization of carrageenans extracted from marine red algae, induced an increase in plant growth (González et al., 2013a) as well as in protection against pathogens (Vera et al., 2011). OCs kappa, lambda and iota are constituted by around 20 units of sulfated galactose linked by alternate β-1,4- and α-1,3-glycosidic bonds with sulfate groups located in positions 2, 4, and 6 of the galactose ring, with or without anhydrogalactose units (for models see Vera et al., 2011). In tobacco plants, stimulation of growth is due to an increase in net photosynthesis, basal metabolism and cell division, mainly in response to OC kappa and iota (Castro et al., 2012). In tobacco plants, OCs increased CO_2_ incorporation, stomatal conductance, PSII efficiency, chlorophyll *a* and *b* content, and rubisco activity indicating an increase in net photosynthesis and C assimilation (Castro et al., 2012). In addition, activities of basal metabolism enzymes producing NAD(P)H were also enhanced in tobacco plants suggesting that the content of reducing power [NAD(P)H] may also be increased (Castro et al., 2012). Moreover, the level of transcripts encoding cyclins A and D (cycA and cycD, respectively), and cyclin-dependent kinases A and B (CDKA and CDKB, respectively), were also increased suggesting a stimulation of cell cycle and cell division (Castro et al., 2012). Furthermore, treatment with OCs increased the level of ASC, but not GSH, which may help the observed increase in cell division in tobacco plants (Castro et al., 2012). In *Eucalyptus globulus* trees, OCs, mainly OC kappa, induced an increase in net photosynthesis probably leading to an increase in NAD(P)H and in the content of cellulose (González et al., 2013b) as well as activities of NAD(P)H-synthesizing enzymes involved in basal metabolism (González, unpublished). Thus, it possible that OC kappa can induce an increase NAD(P)H, ASC, and GSH levels changing the redox status to a more reducing condition that activate TRR/TRX system which may, subsequently, enhance photosynthesis, basal metabolism and growth in *Eucalyptus* trees.

In order to analyze the influence of OC kappa in the redox status of *E. globulus*, the levels of NAD(P)H, ASC and GSH and TRR/TRX activities as well as net photosynthesis, activities of enzymes involved in basal metabolism, and growth were determined in control *Eucalyptus* and in trees treated with OC kappa. In addition, the effects of inhibitors of NAD(P)H, ASC, and GSH syntheses corresponding to CHS-828, lycorine and buthionine sulfoximine (BSO), respectively, as well as an inhibitor of TRR, auranofin, were analyzed in control *Eucalyptus* and in trees treated with OC kappa.

## Materials and methods

### Preparation of OC kappa

OC kappa was prepared from pure commercial kappa2 carrageenan as described in González et al. (2013b).

### Plant culture, treatment with OC kappa, with inhibitors and OC kappa, and measurement of height

*E. globulus* trees with an initial height of 30 cm (*n* = 10 for each group) were cultivated outdoors during austral summer, from December 2012 to March 2013, in plastic bags containing compost (soil supplemented with leaves of Chilean trees). *E. globulus* trees were sprayed in the upper and lower part of the leaves with 5 mL per plant with water/methanol 9:1 v/v (control group 1, *n* = 10), a water/methanol solution of 250 μM CHS-828, an inhibitor of nicotinamide phosphoribosyl transferase (Olessen et al., 2010) and NAD(P)H synthesis (control group 2, *n* = 10), a water/methanol solution of 250 μM lycorine, an inhibitor of GLDH (Arrigoni et al., 1996), and of ASC synthesis (control group 3, *n* = 10), a water/methanol solution of 1.5 mM BSO, an inhibitor of γ-GCS (Griffith and Meister, 1979) and of GSH synthesis (control group 4, *n* = 10), and with auranofin (Gromer et al., 1998), an inhibitor of TRR activity (control group 5, *n* = 10), with OC kappa at a concentration of 1 mg mL^−1^ (treated group 1, *n* = 10), CHS-828 and OC kappa (treated group 2, *n* = 10), lycorine and OC kappa (treated group 3, *n* = 10), BSO and OC kappa (treated group 4, *n* = 10), and auranofine and OC kappa (treated group 5, *n* = 10), and cultivated for 4 months without additional treatment. Trees of control groups 2, 3, 4 and 5 were treated twice with inhibitors, once a week, and then cultivated without additional treatment. Trees of treated groups 2, 3, 4, and 5 were treated twice with inhibitors, once a week, and after 2 weeks they were treated with OC kappa at a concentration of 1 mg mL^−1^, once a week, four times in total and then cultivated for 4 months without additional treatment. Leaves were obtained from the middle part of control and treated trees and pooled into three groups for further analysis (*n* = 3). The height of trees (*n* = 10) was determined using a measuring tape.

It is important to mention that different concentration CHS-828, lycorine, BSO, and auranofin were sprayed on *Eucalyptus* leaves in previous experiments to determine the optimal concentration of each inhibitor (data not shown). In addition, it was determined that the optimal concentration of CHS-828 decreased NADPH content, the optimal concentration of lycorine inhibited galatonolactone dehydrogenase (GLDH) activity, the optimal concentration of BSO inhibited γ-GCS activity, and the optimal concentration of auranofin inhibited TRR activity, up to 4 months of culturing (see Supplementary Fig. 1).

### Determination of NAD(P)H levels

The extraction of reduced pyridine nucleotides was performed as described in Brugidou et al. (1991). Reduced pyridine nucleotides (NADH and NADPH) were extracted from leaves [1 g of fresh tissue (FT)] of control and treated Eucalyptus trees (*n* = 3 for each group). Leaves were frozen in liquid nitrogen and pulverized in a mortar with a pestle. Five mL of 0.5 M HClO_4_ in 10% methanol were added and homogenization was pursued until thawing. The homogenate was filtered through Miracloth (Calbiochem, San Diego, CA). The filtrate was centrifuged at 27,000 *g* for 15 min and the supernatant was recovered. The supernatant was neutralized with 1 M KOH to reach pH = 5.0 and dried in a vacuum evaporator Savant model SpeedVac. The pellet was solubilized in 0.5 mL of 10 mM buffer phosphate pH = 6.5. Oxidized pyridine nucleotides (NAD and NADP) were extracted from leaves (1 g of FT) as described above using 5 mL of 0.5 M NaOH in 10% methanol and the extract neutralized with 1 M HCl to reach pH = 8.0.

Pyridine nucleotides were analyzed by High Performance Liquid Chromatography (HPLC) using an Agilent 1260 Infinity system and data was compiled using OpenLAB software. Pyridine nucleotides (20 μL) were separated on a reversed-phase C-18 column (5 μm particle size, 4.6 mm inner diameter, 15 cm length) at 22°C, eluted using solvent A (50 mM ammonium acetate pH 6.9) and solvent B (100% acetonitrile) with linear step gradients of 25 min from 0 to 5% of solvent B, 7 min from 5 to 95% of solvent B, 3 min from 95 to 100% of solvent B and 10 min of 0% solvent B using a flow rate of 1 mL min^−1^. NAD and NADP were detected by absorbance at 260 nm using a diode array detector and NADH and NADPH using an excitation wavelength of 340 nm and emission wavelength of 480 nm, using a fluorescence detector. Pure NADPH, NADH, NAD, and NADP (Sigma St. Louis, USA) were dissolved in filtered water and used as standards. Retention times of NADPH, NADH, NAD, and NADP were 6.3, 10.7, 31.1, and 32.6 min, respectively.

### Determination of ASC, DHA, GSH, and GSSG levels

The levels of ASC, DHA, GSH, and GSSG were determined as described by Ratkevicius et al. (2003). For detection of ASC/DHA and GSH/GSSG, 1 g of leaves (FT) was used in each case.

### Preparation of protein extracts

*Eucalyptus* leaves (5 g of FT) extracts were prepared as described in Castro et al. (2012) but proteins in extracts were precipitated with ammonium sulfate at 0.6 g mL^−1^ of extract.

### Determination of GLDH and γ-GCS activities

GLDH and γ-GCS activity was determined as described in Mellado et al. (2012).

### Determination of TRR and TRX activities

TRR activity was detected as described in Arnér and Holmgren (2001) using 1 mL of reaction mixture containing 100 mM HEPES buffer pH = 7.6, 0.5 mM dithionitrobenzoic acid (DTNB), 0.3 mM NADPH and 20 μg of protein extract. The increase in absorbance due to thionitrobenzoate (TNB) synthesis was measured at 412 nm for 5 min. TRR activity was calculated using the extinction coefficient of TNB (ε = 14.45 mM^−1^ cm^−1^).

TRX activity was detected as described in Arnér and Holmgren (2001) using 1 mL of reaction mixture containing 100 mM HEPES pH = 7.6, 0.13 mM human insulin, 0.3 mM DTT and 20 μg of protein extract. The increase in turbidity was measured at 650 nm for 3 min. TRX activity was calculated using turbidity coefficient of the large chain of human insulin at 650 nm (ε = 34 mM^−1^ cm^−1^). Regarding TRX activity, it is important to point out that extracts precipitated with ammonium sulfate did contain small molecules such as NADPH that activate TRR, and the reduction of TRR was obtained using DTT.

### Determination of basal metabolism enzyme activities

Rubisco activity was detected as described in Lilley and Walker (1974) using 1 mL reaction mixture containing 100 mM Tris-HCl (pH 8.0), 1 mM ribulose 1,5-biphosphate, 10 mM KHCO_3_, 20 mM MgCl_2_, 5 mM creatine phosphate, 3 mM ATP, 10 U phosphoglycerate kinase, 10 U glyceraldehyde 3-phosphate dehydrogenase, 10 U creatine kinase, 0.15 mM NADH and 10 μg of protein extract. The decrease in absorbance at 340 nm due to consumption of NADH was detected for 3 min. Rubisco activity was calculated using the extinction coefficient of NADH (ε = 6.2 mM^−1^ cm^−1^).

GlnS activity was determined as described in Barbosa et al. (2010) using 1 mL of reaction mixture containing 200 mM HEPES buffer (pH 7.0), 50 mM L-glutamate, 5 mM hydroxylamine, 50 mM magnesium chloride, 20 mM ATP and 100 μg of protein extract. The reaction was incubated at 37°C for 1 h and stopped by addition of 1 mL mixture containing 0.7 M ferric chloride, 20 % (w/v) trichloroacetic acid and 0.3 M HCl. The mixture was centrifuged at 7,400 *g* for 5 min and the supernatant was recovered. The absorbance of the supernatant was detected at 540 nm. GlnS activity was calculated using the extinction coefficient of γ-glutamyl-hydroxamate (ε = 0.85 mM^−1^ cm^−1^).

APR activity was determined as described by Brychkova et al. (2012) using 1 mL in 0.1 mL of reaction mixture containing 70 mM Tris-acetate pH = 8.0, 350 mM sodium sulfate, 300 mM adenosine 5′phosphosulfate (APS), 4.8 mM reduced glutathione (GSH) and 20 μg of protein extract. The reaction mixture was incubated for 30 min at 35°C and an aliquot of 50 μL was added to 450 μL of the coloring mixture containing 340 mM fuchsin, 230 mM sulphuric acid, and 0.1 mM formaldehyde. APR activity was measured by the increase in absorbance at 570 nm due to the formation of a colored complex, and calculated using the extinction coefficient of the colored complex (ε = 40 mM^−1^ cm^−1^).

O-ASTL activity was determined as described in Lunn et al. (1990) using 1 mL of reaction mixture containing 50 mM phosphate buffer pH = 7.5, 10 mM O-acetylserine, 2 mM Na_2_S, 30 mM DTT, and 30 μg protein extract. The reaction was incubated at 37°C for 1 h and the reaction was stopped by addition of 0.5 mL of 20% (w/v) trichloroacetic acid. Cysteine was detected by adding 100 μL of acetic acid and 200 μL of ninhydrin reagent [250 mg of ninhydrin dissolved in 10 mL of concentrated acetic acid: concentrated HCl 60:40 (v/v)]. The mixture was placed in boiling water for 10 min, rapidly cooled in ice and 550 μL of 95% (v/v) ethanol were added. The absorbance of the mixture was determined at 560 nm and O-ASTL activity was determined using the extinction coefficient the spirane formed by cysteine and ninhydrin (ε = 25 mM^−1^ cm^−1^).

Pyruvate dehydrogenase (PDH), isocitrate dehydrogenase parethesis (IDH), 2-oxoglutarate dehydrogenase (OGDH) activities belonging to the Krebs cycle, inosinemonophosphate dehydrogenase (IMPDH), and dihydroorotate dehydrogenase (DHODH) activities involved in purine/pyrimidine synthesis, and glucose 6-P dehydrogenase (G6PDH) activity were determined as described in Castro et al. (2012).

### Statistical analysis

Significant differences were determined by Two-Way analysis of variance (ANOVA) followed by Tukey's multiple comparison tests (*T*). To analyze height, mean values were obtained from ten trees, whereas for the rest of the parameters three independent samples of leaves were used. Differences between mean values were considered to be significant at a probability below 5% (*P* < 0.05), as described by Zar (1999).

## Results

### OC kappa-induced increase in NADPH synthesis requires the increase in ASC synthesis and TRR/TRX activities, but not GSH synthesis

**Figure 1:**
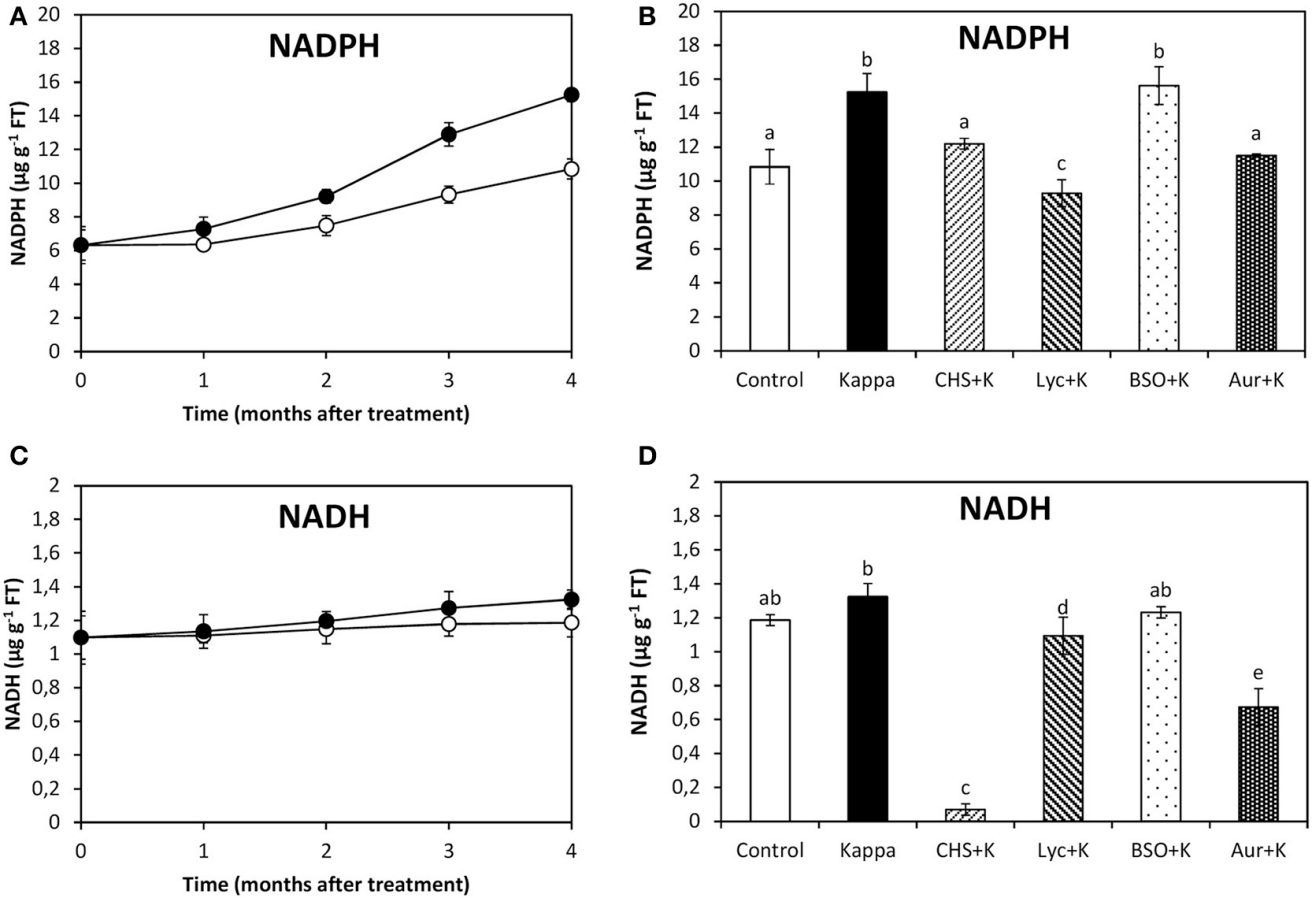
**Effect of inhibitors on NADPH synthesis after treatment with OC kappa**. Levels of NADPH **(A)** and NADH **(C)** in control *Eucalyptus* trees (empty circles), in trees treated with OC kappa (black circles) and cultivated for 0–4 months without additional treatment. Level of NADPH **(B)** and NADH **(D)** in control *Eucalyptus* trees (control), in trees treated with OC kappa (kappa) and in trees treated with CHS-828 and OC kappa (CHS+K), lycorine and OC kappa (Lyc+K), buthionine sulfoximine and OC kappa (BSO+K), and auranofin and OC kappa (Aur+K) and cultivated for 4 months without additional treatment. NAD(P)H levels are expressed in micrograms per gram of fresh tissue (FT). Symbols and bars represent mean values of three independent experiments and letters indicate significant differences (*p* < 0.05).

At 4 months after treatment, NADPH content in control *Eucalyptus* was c. (aproximately) 10.8 μg g^−1^ of FT, and in trees treated with OC kappa it was c. 15.3 μg g^−1^ of FT, corresponding to a c. 42% increase in OC kappa-treated trees in relation to controls (Figure 1A). The increase in NADPH in OC kappa-treated trees was observed to decrease under CHS-828, lycorine, and auranofin, but that was not observed with BSO at 4 months experiments (Figure 1B). In contrast, NADH content did not change significantly under OC kappa treatment (Figure 1C). NADH decreased with CHS-828 and auranofin below levels recorded in controls, decreased in relation to OC kappa-treated by lycorine, but did not change with BSO (Figure 1D).

### OC-kappa-induced increase in ASC synthesis requires the increase in NADPH synthesis and TRR/TRX activities, but not GSH synthesis

**Figure 2:**
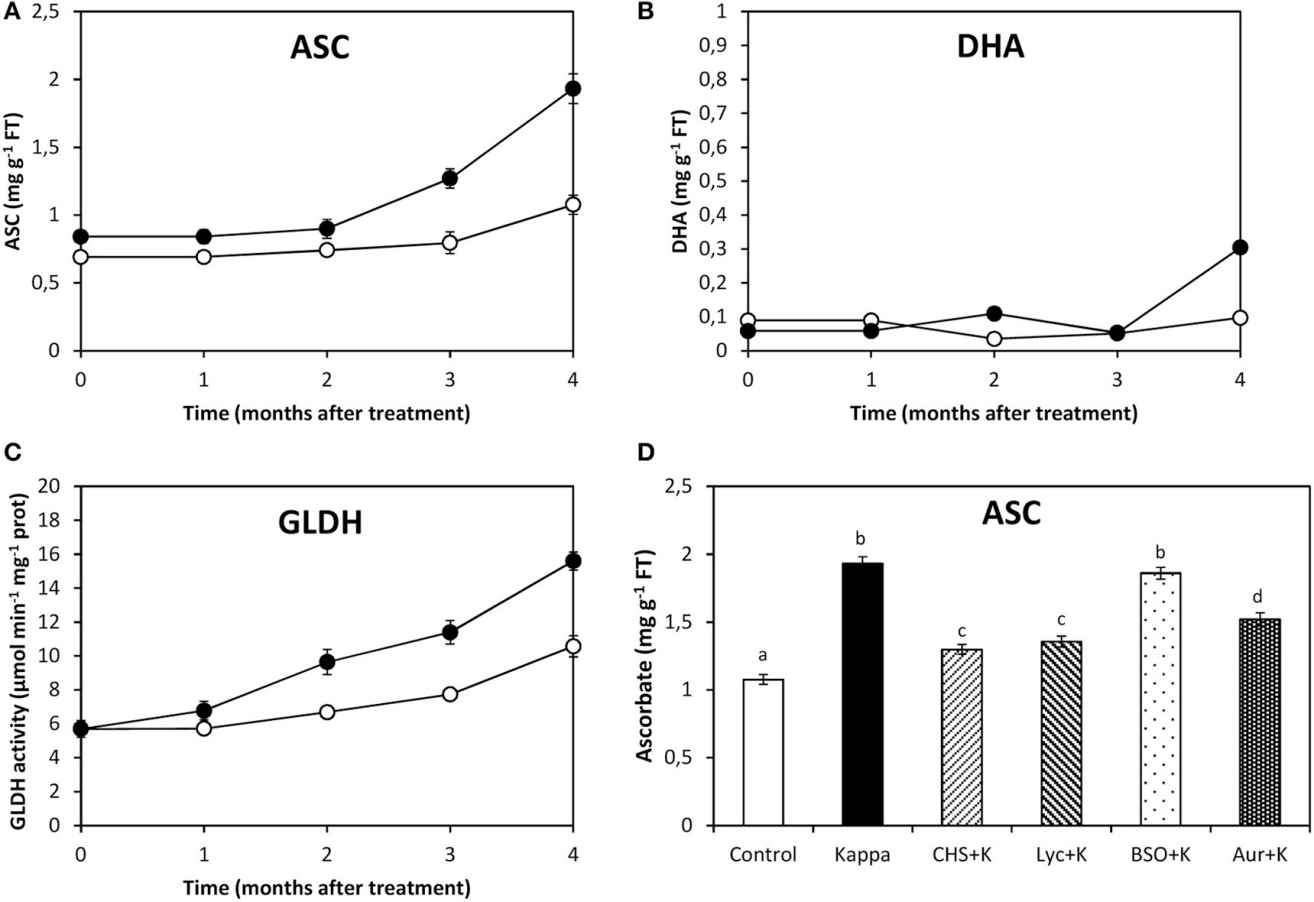
**Effect of inhibitors on ASC synthesis after treatment with OC kappa**. Level of ascorbate [ASC, **(A)**] and dehydroascorbate [DHA, **(B)**], and galatonolactone dehydrogenase [GLDH, **(C)**] activity in control *Eucalyptus* trees (empty circles) and in trees treated with OC kappa (black circles) and cultivated for 0–4 months without additional treatment. Level of ASC **(D)** in control *Eucalyptus* trees (control), in trees treated with OC kappa (kappa) and in trees treated with CHS-828 and OC kappa (CHS+K), lycorine and OC kappa (Lyc+K), buthionine sulfoximine and OC kappa (BSO+K) and auranofin and OC kappa (Aur+K) and cultivated for 4 months without additional treatment. ASC and DHA levels are expressed in milligrams per gram of fresh tissue (FT) and the activity of GLDH is expressed in micromoles per minute per milligram of protein. Symbols and bars represent mean values of three independent experiments and letters indicate significant differences (*p* < 0.05).

After 4 months treatment, ascorbate (ASC) content in control *Eucalyptus* was c. 1.1 mg g^−1^ FT, whereas in trees treated with OC kappa it was c. 1.9 mg g^−1^ FT, corresponding to a c. 73% increase in OC kappa-treated trees in relation to controls (Figure 2A). Dehydroascorbate (DHA) content in control trees was c. 0.1 mg g^−1^ FT and in trees treated with OC kappa it was c. 0.3 mg g^−1^ FT, which corresponds to about 3-fold increase (Figure 2B). The activity of GLDH, the last and regulatory enzyme of ASC synthesis, was 32 μmoles min^−1^ mg^−1^ protein in controls, while in OC kappa-treated trees it was 53 μmoles min^−1^ mg^−1^ protein, corresponding to a c. 66% increase (Figure 2C). ASC synthesis decreased after treatment with CHS-828, lycorine and auranofin to c. 18, 27, and 36%, respectively, but did not change with BSO (Figure 2D).

### OC-kappa-induced increase in GSH synthesis requires the increase in NADPH synthesis and TRR/TRX activities, but not ASC synthesis

**Figure 3:**
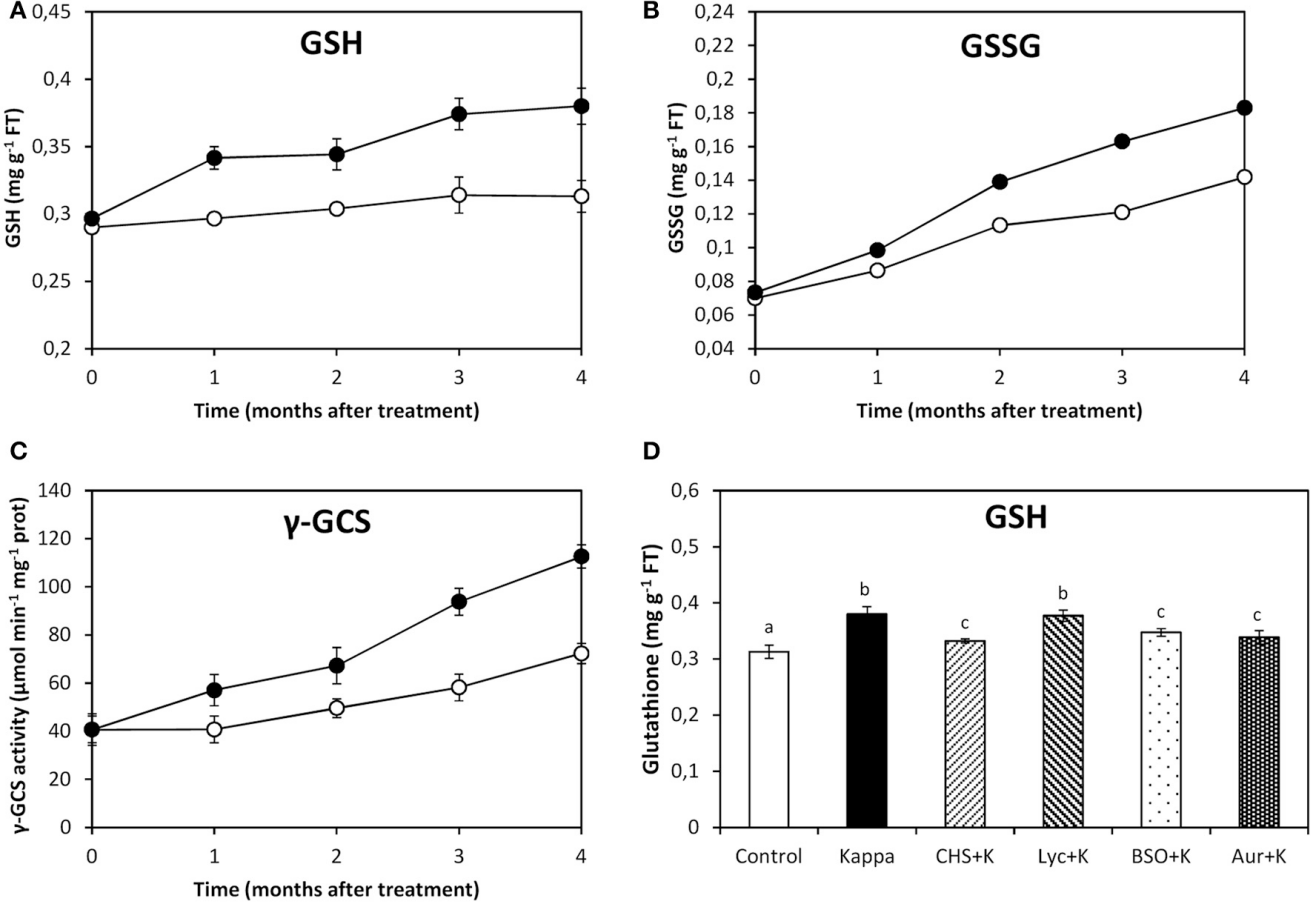
**Effect of inhibitors on GSH synthesis after treatment with OC kappa**. Level of glutathione [GSH, **(A)**] and oxidized glutathione [GSSG, **(B)**] and of γ-glutamylcysteine synthase [γ-GCS, **(C)**] activity in control *Eucalyptus* trees (empty circles) and in trees treated with OC kappa (black circles) cultivated for 0–4 months without additional treatment. Level of GSH **(D)** in control *Eucalyptus* trees (control), in trees treated with OC kappa (kappa) and in trees treated with CHS-828 and OC kappa (CHS+K), lycorine and OC kappa (Lyc+K), buthionine sulfoximine and OC kappa (BSO+K) and auranofin and OC kappa (Aur+K) and cultivated for 4 months without additional treatment. GSH and GSSG levels are expressed in milligrams per gram of fresh tissue (FT) and the activity of γ-GCS is expressed in micromoles per minute per milligram of protein. Symbols and bars represent mean values of three independent experiments and letters indicate significant differences (*p* < 0.05).

At 4 months after treatment, glutathione (GSH) content in control *Eucalyptus* was c. 0.3 mg g^−1^ FT and in OC kappa-treated trees was c. 0.4 mg g^−1^ FT, corresponding to a c. 33% increase (Figure 3A). Oxidized glutathione (GSSG) content in control trees was 0.1 mg g^−1^ FT and in trees treated with OC kappa it was 0.2 mg g^−1^ FT, which is 2-fold increase in relation to controls (Figure 3B). The activity of γ-GCS, the first and regulatory enzyme of GSH synthesis, was c. 72 μmoles min^−1^ mg^−1^ protein in control trees and c. 112 μmoles min^−1^ mg^−1^ protein OC kappa-treated trees, a c. 57% (Figure 3C). In relation to OC kappa treatment, there was a decrease in GSH levels in c. 33% under CHS-828, BSO, and auranofin, but not with lycorine (Figure 3D).

### OC-kappa-induced increase in TRR/TRX requires the increase in NADPH synthesis, but not ASC and GSH syntheses

**Figure 4:**
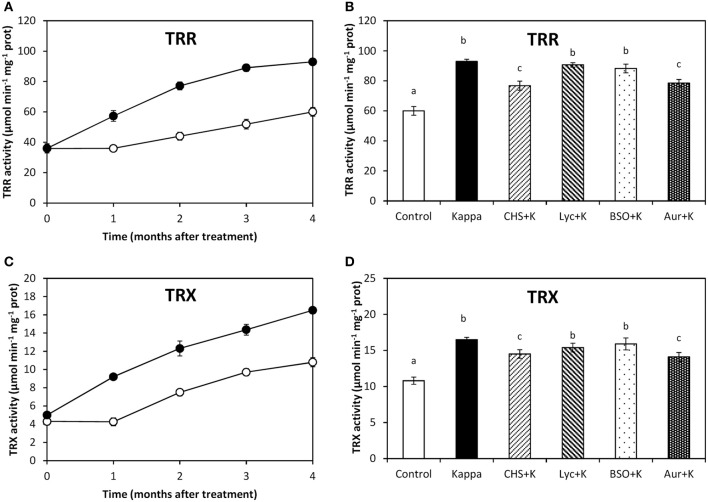
**Effect of inhibitors on TRR and TRX activities after treatment with OC kappa**. Activities of thioredoxin reductase [TRR, **(A)**] and thioredoxin [TRX, **(C)**] in control *Eucalyptus* trees (empty circles) and in trees treated with OC kappa (black circles) cultivated for 0–4 months without additional treatment. Activities of TRR **(B)** and TRX **(D)** in control *Eucalyptus* trees (control), in trees treated with OC kappa (kappa) and in trees treated with CHS-828 and OC kappa (CHS+K), lycorine and OC kappa (Lyc+K), buthionine sulfoximine and OC kappa (BSO+K) and auranofin and OC kappa (Aur+K) and cultivated for 4 months without additional treatment. TRR and TRX activities are expressed in micromoles per minute per milligram of protein. Symbols and bars represent mean values of three independent experiments and letters indicate significant differences (*p* < 0.05).

TRR activity in control *Eucalyptus* trees was c. 60 μmoles min^−1^ mg^−1^ protein and in trees treated with OC kappa it was 93 μmoles min^−1^ mg^−1^ protein, at 4 months after treatment, corresponding to a c. 55% increase (Figure 4A). At 4 months after treatment, TRR activity decreased in relation to OC kappa treatment under CHS-828 and auranofin to c. 28 and c. 32%, respectively, but not with lycorine or BSO (Figure 4B). In addition, TRX activity in control trees was c. 11 μmoles min^−1^ mg^−1^ protein and in OC kappa-treated trees was c. 17 μmoles min^−1^ mg^−1^ protein, corresponding to a c. 55% increase (Figure 4C). TRX activity decreased under CHS-828 and auranofin in c. 39% and c. 30%, respectively, but that was not observed with lycorine or BSO (Figure 4D).

### OC kappa-induced increase in height and photosynthesis requires the increase in NADPH, ASC, GSH syntheses, and TRR/TRX activities

**Figure 5:**
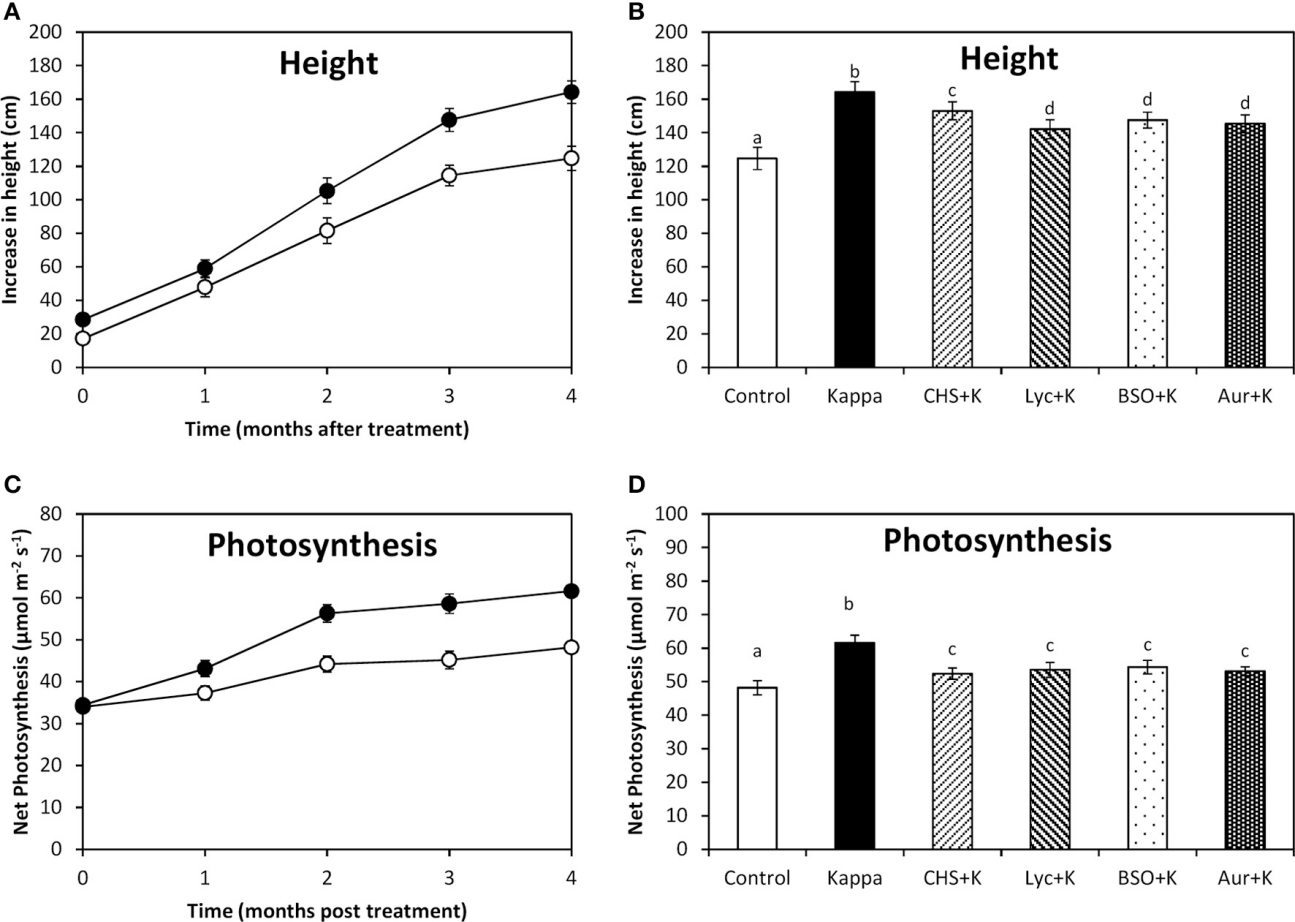
**Effect of inhibitors on height and photosynthesis after treatment with OC kappa**. Increase in height **(A)** and net photosynthesis **(C)** in control *Eucalyptus* trees (empty circles), in trees treated with OC kappa (black circles) and cultivated for 0–4 months without additional treatment. Increase in height **(B)** and net photosynthesis **(D)** in control *Eucalyptus* trees (control), in trees treated with OC kappa (kappa) and in trees treated with CHS-828 and OC kappa (CHS+K), lycorine and OC kappa (Lyc+K), buthionine sulfoximine and OC kappa (BSO+K) and auranofin and OC kappa (Aur+K) and cultivated for 4 months without additional treatment. The increase in height is expressed in centimeters and net photosynthesis in micromoles per meter square per second. Symbols and bars represent mean values of three independent experiments and letters indicate significant differences (*p* < 0.05).

At 4 months after treatment, height of control *Eucalyptus* trees increased in c. 125 cm, whereas OC kappa-treated trees the increase was c. 164 cm, which corresponds to an increase of c. 31 % compared to controls (Figure 5A). The increase in height observed in OC kappa-treated trees for 4 months was inhibited by CHS-828, lycorine, BSO, and auranofin, in c. 22, 14, 17, and 16%, respectively (Figure 5B). Net photosynthesis in control *Eucalyptus* was c. 48 μmoles m^−2^ s^−1^ and in trees treated with OC kappa for 4 months it was c. 62 μmoles m^−2^ s^−1^, which corresponds to an increase of c. 29% compared to controls (Figure 5C). The increase in net photosynthesis was inhibited by CHS-828, lycorine, BSO and auranofin in c. 9, 12, 12, and 10%, respectively (Figure 5D).

### OC-kappa increase in basal metabolism requires the increase in NADPH, ASC and GSH syntheses, and TRR/TRX activities

**Figure 6:**
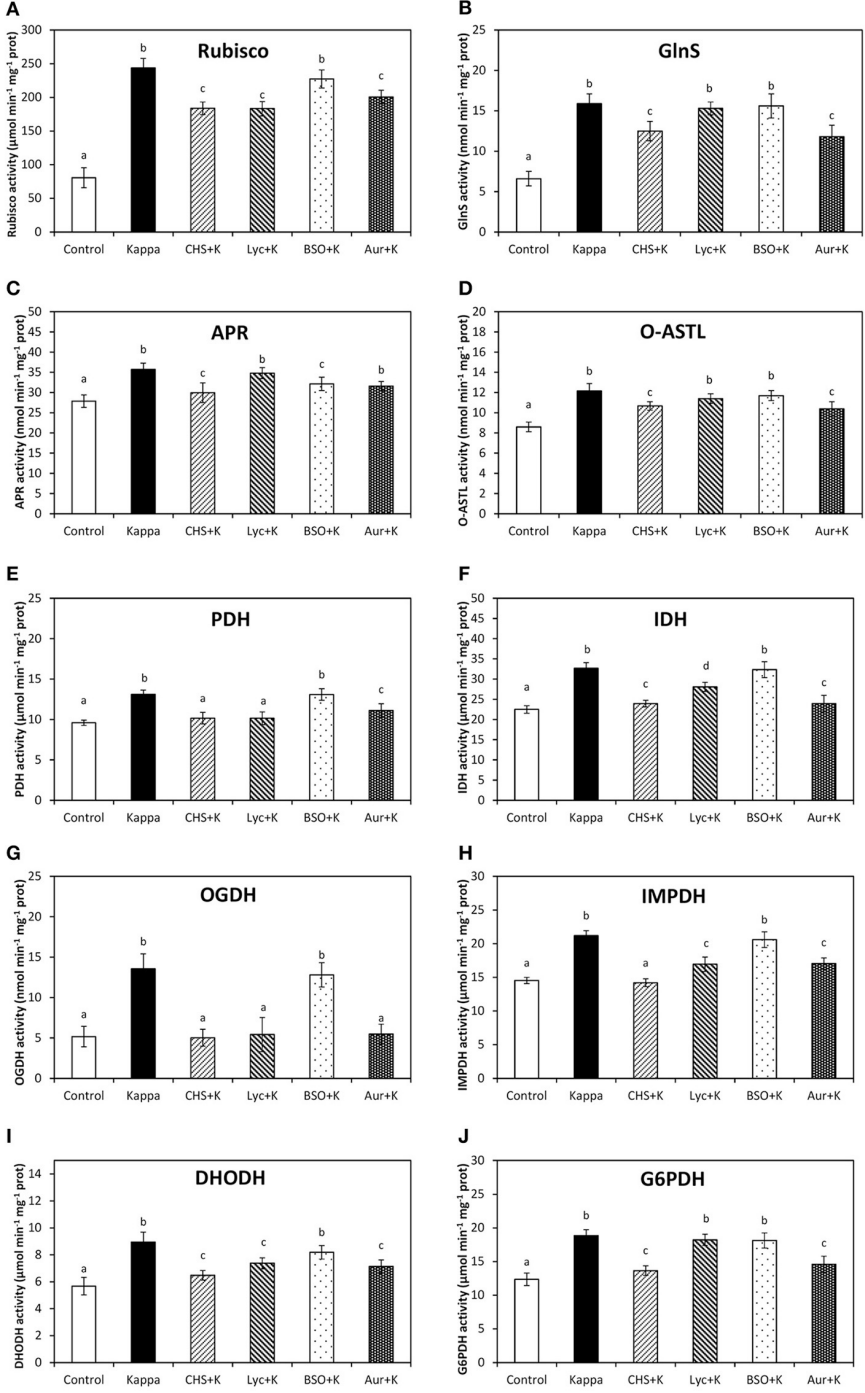
**Effect of inhibitors on basal metabolism enzymes activities after treatment with OC kappa**. Activities of rubisco **(A)**, glutamine synthetase [GlnS, **(B)**], adenosine 5′phosphosulfate reductase [APR, **(C)**], O-acetylserinethiol-lyase [O-ASTL, **(D)**], pyruvate dehydrogenase [PDH, **(E)**], isocitrate dehydrogenase [IDH, **(F)**], 2-oxoglutarate dehydrogenase [OGDH, **(G)**], inosine monophosphate dehydrogenase [IMPDH, **(H)**], dihydroorotate dehydrogenase [DHODH, **(I)**], and glucose 6-phospahte dehydrogenase [G6PDH, **(J)**] in control *Eucalyptus* trees (control), in trees treated with OC kappa (kappa) and in trees treated with CHS-828 and OC kappa (CHS+K), lycorine and OC kappa (Lyc+K), buthionine sulfoximine and OC kappa (BSO+K) and auranofin and OC kappa (Aur+K) and cultivated for 4 months without additional treatment. Activities of rubisco, O-ASTL PDH, IDH, OGDH, IMPDH, DHODH, and G6PDH are expressed in micromoles per minute per milligram of protein and activities of GlnS and APR are expressed in nanomoles per minute per milligram of protein. Bars represent mean values of three independent experiments and letters indicate significant differences (*p* < 0.05).

Rubisco activity, the first and regulatory enzyme of C assimilation, in control *Eucalyptus* was c. 81 μmoles min^−1^ mg^−1^ protein and in OC kappa-treated trees was c. 244 μmoles min^−1^ mg^−1^ protein at 4 months after treatment, corresponding to a 3-fold increase. Rubisco activity decreased under CHS-828, lycorine, auranofin treatments c. 1.3, c. 1.3, and c. 1.5 times, but that was not observed with BSO (Figure 6A). At 4 months after treatment, GlnS activity, the regulatory enzyme of N assimilation, was c. 7 μmoles min^−1^ mg^−1^ protein in control trees and in OC kappa-treated trees was c. 16 μmoles min^−1^ mg^−1^ protein, corresponding to a c. 2-fold increase (Figure 6B). GlnS activity decreased under CHS-828 and auranofin c. 1.8 and 1.7 times, respectively, but no significant change was recorded with lycorine or BSO. APR activity, the regulatory enzyme of S assimilation, was 28 μmoles min^−1^ mg^−1^ protein in control *Eucalyptus* and in OC kappa-treated trees was c. 36, corresponding to a c. 29% increase. APR activity decreased under CHS-828, auranofin and BSO treatments in c. 7, 14, and 14%, respectively, although no significant changes were recorded with lycorine (Figure 6C). O-ASTL activity, the last enzyme for S assimilation, in control *Eucalyptus* trees was c. 9 μmoles min^−1^ mg^−1^ protein while in OC kappa-treated trees was c. 12 μmoles min^−1^ mg^−1^ protein at 4 months after treatment, corresponding to a c. 33% increase. O-ASTL activity decreased under CHS-828 and auranofin treatments to c. 22 and 11%, but no change was observed with lycorine or BSO (Figure 6D).

PDH activity, the first enzyme of the Krebs cycle, was 10 μmoles min^−1^ mg^−1^ protein in control *Eucalyptus* and in OC kappa-treated trees was 13 μmoles min^−1^ mg^−1^ protein at 4 months after treatment, corresponding to a c. 30% increase. PDH activity decreased under CHS-828 and lycorine and auranofin treatments, although in the latter the decrease was more moderated, but in case of BSO treatment no significant changes were observed with respect to OC kappa-treated trees (Figure 6E). IDH, the fourth enzyme of the Krebs cycle, was c. 23 μmoles min^−1^ mg^−1^ protein in control *Eucalyptus* and in OC kappa-treated trees was c. 33 μmoles min^−1^ mg^−1^ protein at 4 months after treatment, corresponding to a c. 43% increase. IDH activity decreased under CHS-828, lycorine and auranofin in c. 4, 22, and 4%, respectively, but no significant changes were recorded with BSO (Figure 6F). At 4 months after treatment, 2-oxoglutarate dehydrogenase (OGDH) activity, the fifth enzyme of the Krebs cycle, was c. 5 μmoles min^−1^ mg^−1^ protein in control *Eucalyptus* and in OC kappa-treated trees was c. 14 μmoles min^−1^ mg^−1^ protein, almost a 3-fold increase. OGDH activity decreased under CHS-828, auranofin, and lycorine treatments, but not with BSO (Figure 6G).

Inosine monophosphate dehydrogenase (IMPDH) activity, involved in purine synthesis, in control *Eucalyptus* was c. 15 μmoles min^−1^ mg^−1^ protein and in OC kappa-treated was c. 21 μmoles min^−1^ mg^−1^ protein, corresponding to a c. 40% increase. IMPDH activity markedly decreased under CHS-828, moderately decreased in c. 13% under auranofin and lycorine, but was not recorded to decrease with BSO (Figure 6H). DHODH activity, involved in pyrimidine synthesis, was c. 6 μmoles min^−1^ mg^−1^ protein in control *Eucalyptus* and c. 9 μmoles min^−1^ mg^−1^ protein in OC kappa-treated trees, resultant in a c. 50% increase. DHODH activity decreased under CHS-828, auranofin and lycorine in c. 17% in each case, but no significant differences were observed under BSO (Figure 6I). At 4 months after treatment, G6PDH activity, involved in ribose 5-P synthesis, was c. 12 μmoles min^−1^ mg^−1^ protein in control *Eucalyptus* and c. 19 μmoles min^−1^ mg^−1^ protein in OC kappa-treated trees, corresponding to a c. 58% increase. G6PDH activity decreased in CHS-828 and auranofin treatments in c. 17 and 25%, respectively, but no differences were recorded in lycorine and BSO treatments (Figure 6J).

## Discussion

### OC-kappa-induced increases in NADPH, ASC, GSH syntheses, and TRR/TRX activities are cross-talking events

**Figure 7:**
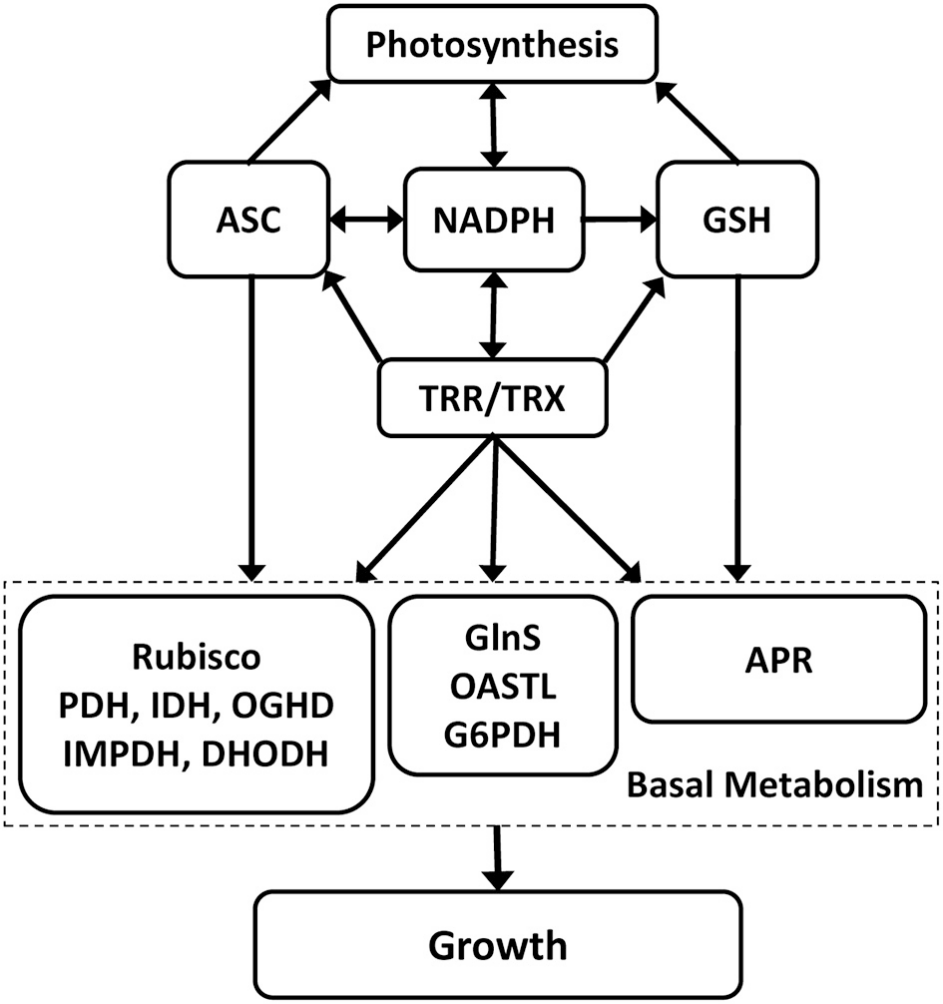
**Model of the interdependence in the increases of NADPH, ascorbate (ASC), glutathione (GSH) syntheses, and TRR (thioredoxin reductase)/TRX (thioredoxin) activities and their interaction with photosynthesis, basal metabolism, and growth**.

In this work, we showed that OC kappa induced a concomitant increase in NADPH, ASC, and GSH syntheses and TRR/TRX activities in *Eucalyptus* trees and that these increases are cross-talking events. Interestingly, the increase in NADPH was influenced by the increase in ASC and GSH and TRR/TRX activities, but, in contrast, the increase in ASC was influenced only by the increase in NADPH-TRR/TRX, and not by the increase in GSH (see model in Figure 7). In addition, the increase in TRR/TRX activities was only influenced by the increase in NADPH, and not by the increase in ASC and GSH. On the other hand, the increase in ASC, GSH, NADPH, and TRR/TRX activities influenced photosynthesis and growth as well as the activity of several basal metabolism enzymes. However, some basal metabolism enzymes were only influenced by the increase in TRR/TRX activities, and not by the increase in ASC or GSH. Thus, despite that ASC and GSH influenced NADPH synthesis, it appears that NADPH is the heart of the redox status since it directly activates TRR/TRX system which, in turn, activates photosynthesis, basal metabolism and growth in *Eucalyptus* trees. Surprisingly, only NADPH increased in response to OC kappa and not NADH. Considering that NAD is the precursor of NADP, NAD might have been converted into NADP and then in NADPH. In this sense, it has been observed that NADPH content is higher than NADH in wheat and pea leaves because the NAD kinase, the enzyme that converts NAD in NADP, is mainly located in chloroplast and is light-dependent (Muto et al., 1981). Thus, a similar phenomenon might occur in leaves of *Eucalyptus* trees treated with OC kappa. In addition, the increase in NADPH synthesis was regulated by TRR/TRX activities suggesting that TRXs participate in the conversion of NAD in NADP. In this regard, it has been observed that NAD kinase is activated by TRXs in *Lycopersicon pimpinellifolium* (Delumeau et al., 2000) and, thus, a similar process may be occurring in *Eucalyptus* trees treated with OC kappa. Moreover, the increase in ASC participates in the increase in NADPH suggesting that ASC may protect photosystems and redox-sensitive proteins in chloroplasts (see below), thus, enhancing photosynthesis that produces NADPH. On the other hand, NADPH-TRR/TRX system induced the increase in ASC and GSH syntheses. Until now, there is no evidence indicating that TRXs bind to enzymes involved in ASC or GSH syntheses (Montrichard et al., 2009). However, TRXs bind to phosphoglucomutase (PGM) in the cyanobacteria *Synechocystis* sp. (Lindhal and Florencio, 2003), an enzyme that converts glucose-1-P into glucose-6-P. Considering that glucose-6-P is a precursor for ASC synthesis (Valpuesta and Botella, 2004), it is possible that the latter is indirectly activated by TRXs through the interaction with PGM. In addition, TRXs activate GluS involved in glutamate synthesis in the green microalga *Chlorella sorokiniana* (Tischner and Schmidt, 1982), and TRXs bind to a cyclophylin that activates O-ASTL, which is involved in cysteine synthesis in *Arabidopsis* (Dominguez-Solís et al., 2008). Considering that GSH is constituted by glutamate and cysteine, it is possible that TRXs may interact with enzymes involved in GSH synthesis and, thus, indirectly increasing GSH synthesis.

### OC kappa-induced increase in ASC, GSH, and NADPH syntheses and TRR/TRX activities, enhance photosynthesis and growth

Our results showed that OC kappa induced an increase in net photosynthesis and growth which were influenced by the increase in ASC, GSH, and NADPH syntheses and TRR/TRX activities in *Eucalyptus* trees. Regarding the increase in ASC and photosynthesis, it is important to mention that ASC is the substrate of the antioxidant enzyme AP that detoxifies hydrogen peroxide produced by electron transport chains (ETCs) in chloroplasts and mitochondria (Shigeoka et al., 2002). In addition, it has been shown that hydrogen peroxide oxidize sulfhydryl groups of redox-sensitive proteins such as rubisco, RAC, and other Calvin cycle enzymes leading to their inactivation and further degradation (García-Ferris and Moreno, 1993; Moreno et al., 2008). Moreover, *Arabidopsis* mutants having a lower ASC content than wild type plants showed degradation of grana stacks in chloroplasts (Olmos et al., 2006). Thus, ASC protects proteins involved in photosynthesis and C assimilation. Furthermore, ASC also regulates cell division in plants since it allows the transition from G1 to S phase in tobacco cells cultured *in vitro* and in cells of the quiescent center in onion roots (Liso et al., 1988; De Pinto et al., 1999). Indeed, *Arabidopsis* mutant *vtc-1* having 70% less ASC in the leaves showed a slower growth rate, smaller leaves and lighter shoots compared to wild-type plants (Veljovic-Jovanovic et al., 2001). Thus, the increase in ASC synthesis observed in *Eucalyptus* trees treated with OC kappa may provide protection of redox-sensitive enzymes and photosystems leading to an increase in photosynthesis which, added to the ASC-dependent stimulation of cell division may determine, at least in part, the increase in growth.

Regarding the increase in GSH and photosynthesis, it has been recently recorded that cucumber plants treated with a brassinosteroid showed an increase in GSH/GSSG ratios, in activities of rubisco and other Calvin-Benson cycle enzyme activities as well as in the content of RAC (Jiang et al., 2012). In addition, it has been observed that GSH is required for transition from G1 to S phase in tobacco cells cultured *in vitro* and cell division in the apical meristems of *Arabidopsis* roots (Vernoux et al., 2000). Furthermore, *Arabidopsis* mutants having a mutation in the gene encoding γ-GCS and in a NADPH-dependent TRR showed a smaller size of shoots and roots (Bashandi et al., 2010). Thus, the increase in GSH levels observed in *Eucalyptus* trees treated with OC kappa may participate in the increase of photosynthesis, which added to the GSH-dependent increase in cell division may determine, at least in part, the increase in growth.

Regarding the increase in NADPH-TRR/TRX system and photosynthesis, it has been shown that TRXs interact with protein D1 protecting PSII from oxidative damage in *Arabidopsis* (Ströher and Dietz, 2008) and with subunits of chloroplast ATPase in *Chlamydomonas reinhardtii* (Lemaire et al., 2004). Moreover, Wang et al. (2013) observed that TRXs-silenced *Arabidopsis* evidenced elevated content of reactive oxygen species and high levels of oxidized subunit CP47 in PSII, explaining the pale green aspect of leaves. In addition, TRX *f* and TRX *m* increased ATPase activity of the subunit CHLI of magnesium chelatase leading to an increase in chlorophyll content and photosynthesis in pea plants (Luo et al., 2012). Therefore, the increase in NADPH synthesis and TRR/TRX activities observed in *Eucalyptus* trees treated with OC kappa may increase chlorophyll synthesis and protection of photosystems leading to an increase in photosynthesis which may determine, at least in part, the increase in growth.

### OC-kappa-induced increase in NADPH, ASC, GSH syntheses, and TRR/TRX activities enhance basal metabolism

In addition, it was observed that the increase in ASC, GSH, and NADPH syntheses and TRR/TRX activities participate in the increase in nutrient assimilation since the increase in ASC, NADPH, and TRR/TRX activities enhanced rubisco activity, the increase in GSH synthesis and TRR/TRX activities enhanced APR activity, and the increase in NADPH synthesis and TRR/TRX activities enhanced GlnS and O-ASTL activities (see model in Figure 7). Regarding rubisco activity and ASC, it is well known that ASC protects rubisco and RAC from oxidation of in their sulfhydryl groups, avoiding their inactivation and degradation (García-Ferris and Moreno, 1993; Moreno et al., 2008). In contrast, rubisco activity was not regulated by GSH content in OC kappa-treated *Eucalyptus* trees, in disagreement with Jiang et al. (2012), which observed that an increase in GSH/GSSG ratios provides protection of rubisco, Calvin cycle enzymes, and RAC from oxidation and degradation. In this sense, it is important to mention that GSH/GSSG ratios did not increase in *Eucalyptus* trees treated with OC kappa (see Supplementary Fig. 2), which would provide explanation for this apparent inconsistency. Regarding the increase in rubisco activity and TRXs, it has been shown that TRXs induce an increase RAC activity which, in turn, activates rubisco in *Arabidopsis* (Zhang and Portis, 1999). Regarding the increase in GSH and APR activity, it is not surprising that GSH enhanced APR activity since GSH is a substrate of this enzyme (Vauclaire et al., 2002). Regarding the increase in GlnS and O-ASTL actitvities and NADPH-TRR/TRX system, it has been observed that GlnS is activated by TRXs in the green microalga *Chlorella sorokiniana* (Tischner and Schmidt, 1982), and that O-ASTL activated by a cyclophylin in *Arabidopsis* that is, in turn, is activated by a TRX (Dominguez-Solís et al., 2008). Thus, the increase in ASC, GSH and NADPH syntheses and TRR/TRX activities enhanced C, N and S assimilation in *Eucalyptus* trees treated with OC kappa, indicating that a reducing redox status favors nutrient assimilation.

In addition, the increase in NADPH and ASC syntheses and TRR/TRX activities enhanced Krebs cycle enzyme activities PDH, IDH, and OGDH. The regulation of Krebs cycle enzymes by ASC suggest that these enzymes are redox-sensitive, as it has been previously shown for PDH and OGDH in *Arabidopsis* (Sweetlove et al., 2002). Moreover, purine and pyrimidine synthesis enzymes IMPDH and DHODH, respectively, were also influenced by ASC and NADPH-TRR/TRX in OC kappa-treated trees. In this sense, there is evidence suggesting that TRXs participate in regulating DHODH since it has been observed that TRXs bind to DHODH in barley (Hägglund et al., 2008). No information is now available regarding the activation of IMPDH by TRXs. In contrast, G6PDH, which produces ribose 5-P, was only regulated by NADPH-TRR/TRX system, which is in agreement with the findings by Née et al. (2009) that observed the activation of G6PDH by TRXs in *Arabidopsis*. Thus, the increase in ASC, GSH NADPH syntheses, and TRR/TRX activities enhanced basal metabolism in *Eucalyptus* trees explaining, at least in part, the increase in growth induced by OC kappa (see model in Figure 7).

Although different investigations have demonstrated that OCs stimulate photosynthesis, basal metabolism, and growth, to date, there is no published information on molecular mechanisms behind these events. Hereby, we determined that OC kappa changed the redox status to a more reducing condition favoring an increase of photosynthesis, nutrients assimilation, basal metabolism, and growth. In addition, it is possible to imagine that OC kappa binds to a specific receptor located in the plasma membrane, inducing signal transduction and the activation of a master protein capable of regulating basal metabolism and photosynthesis. Such a master protein could correspond to the kinase Target of Rapamycin (TOR), a major regulator of nutrient assimilation and basal metabolism in humans, yeast, and plants (Xiong and Sheen, 2014). However, the potential involvement of TOR in the activation of nutrient assimilation and basal metabolism in *Eucalyptus* trees treated with OC kappa remains to be determined.

## Conclusions

In this work, we showed that: (i) OC kappa induced an increase in NADPH, ASC, and GSH syntheses changing the redox status to a more reducing condition, (ii) the increase in NADPH, ASC, and GSH synthesis and TRR/TRX activities are cross-talking events (iii) the increase in NADPH synthesis activate TRR/TRX system, and (iv) the increase in NADPH, ASC, and GSH syntheses and TRR/TRX activities determine, at least in part, the increase in photosynthesis, basal metabolism and growth induced by OC kappa in *Eucalyptus* trees.

### Conflict of interest statement

The authors declare that the research was conducted in the absence of any commercial or financial relationships that could be construed as a potential conflict of interest.
