# Supplementary material for: Oligo-carrageenan kappa increases NADPH, ascorbate and glutathione syntheses and TRR/TRX activities enhancing photosynthesis, basal metabolism, and growth in Eucalyptus trees
Source: Front Plant Sci. 2014 Oct 13;5:512. doi: 10.3389/fpls.2014.00512 (PMC4195311; doi:10.3389/fpls.2014.00512)
Supplement: Supplementary file 1 [file Presentation1.PDF]

SUPPLEMENTARY MATERIAL

Oligo-carrageenan kappa increases NADPH, ascorbate and glutathione syntheses and TRR/TRX activities enhancing photosynthesis, basal metabolism, and growth in *Eucalyptus* trees

Alberto González, Fabiola Moenne, Melissa Gómez, Claudio A. Sáez, Rodrigo A. Contreras and Alejandra Moenne

Marine Biotechnology Laboratory, Department of Biology, Faculty of Chemistry and Biology, University of Santiago of Chile, 917002, Santiago, Chile.

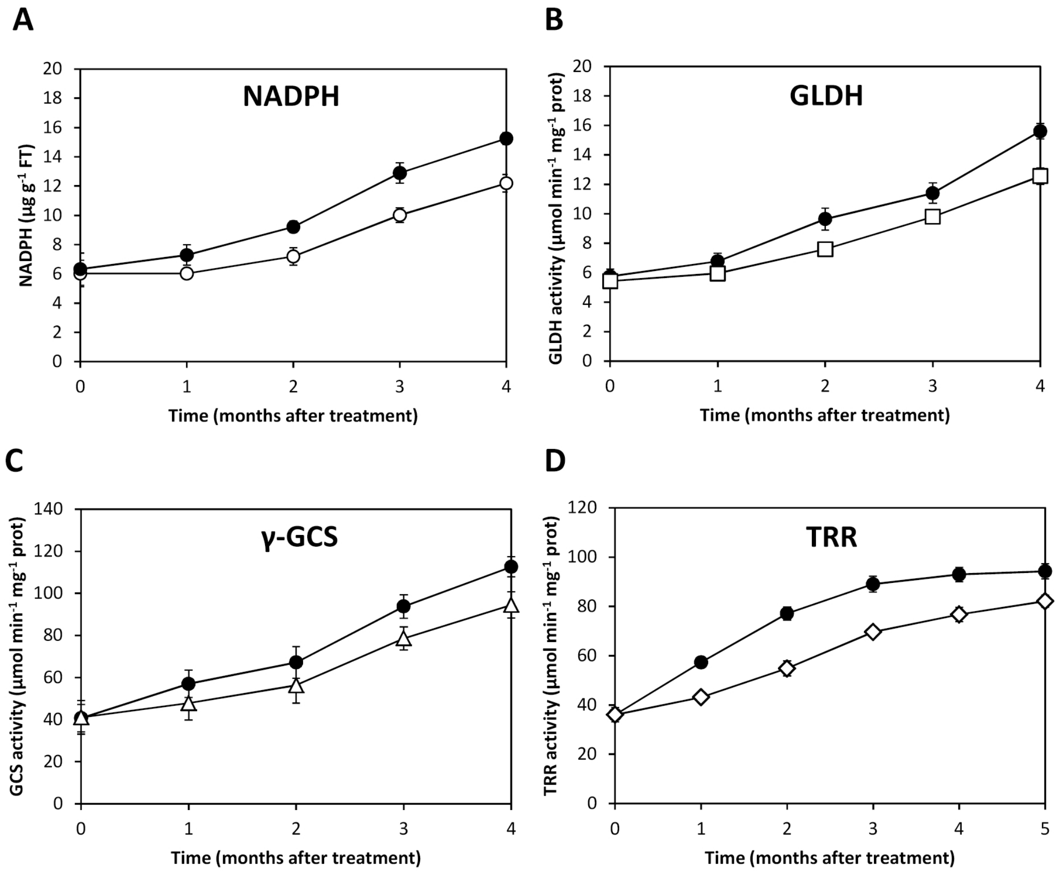

Fig. S1

Figure S1. **Effect of inhibitors on trees treated with OC kappa**. Level of NADPH (A) and activities of galactonolactone dehydrogenase (GLDH, B), γ-glutamylcysteine synthase (γ-G CS, C) and thioredoxin reductase (TRR, D) in control *Eucalyptus* (empty circle), in trees treated with OC kappa (black circle) and in trees treated with auranofin and OC kappa (asterisks), with lycorine and OC kappa (empty squares), with buthionine sulfoximine and OC kappa (empty triangles) and with auranofin and OC kappa (empty diamonds) and cultivated for 0 to 4 months without additional treatment. The level of NADPH is expressed in micrograms per gram of fresh tissue (FT) and the activities of GLDH, γ-GCS and TRR are expressed in micromoles per minute per milligram of protein. Symbols represent mean values of three independent experiments and letters indicate significant differences (p<0.05).

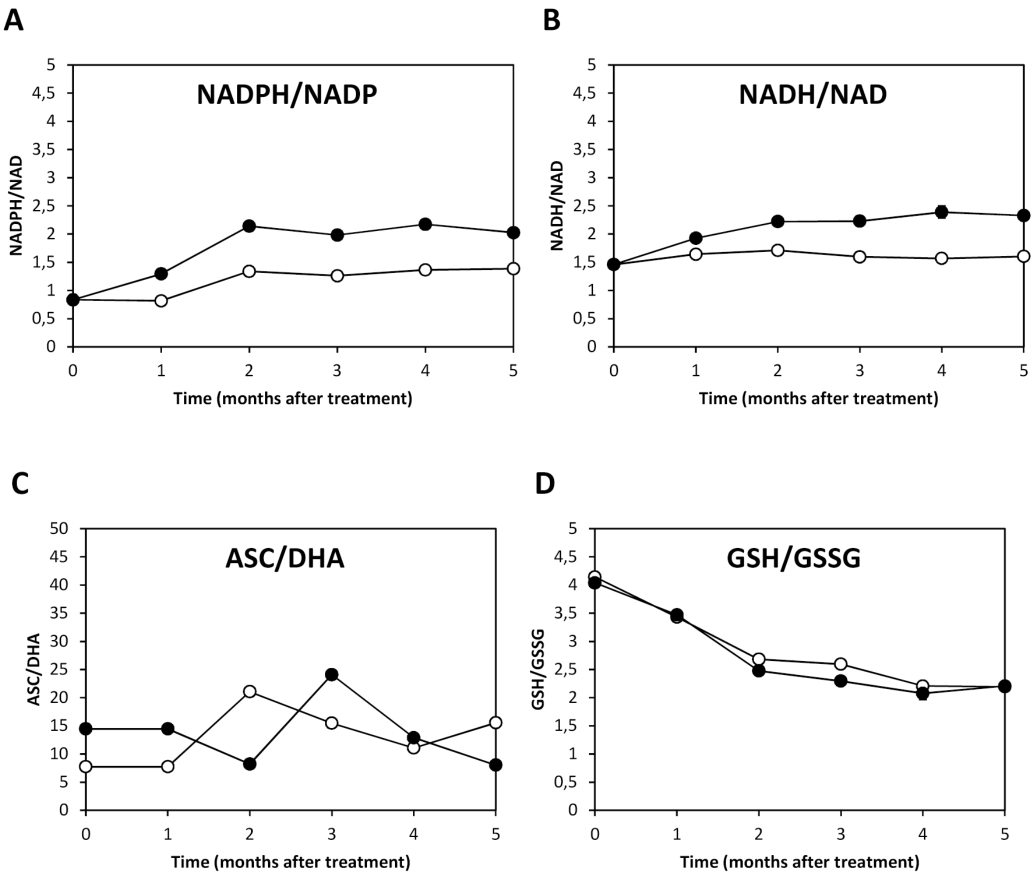

Fig. S2

Figure S2. **Ratios of ASC/DHA and GSH/GSSG in trees treated with OC kappa**. ASC/DHA (A) and GSH/GSSG (B) ratios in control *Eucalyptus* trees (empty circles) and in trees treated with OC kappa (black circles) and cultivated for 0 to 4 months without additional treatment.
